# A Year in Review on Tuberculosis and Non-tuberculous Mycobacteria Disease: A 2026 Update for Clinicians and Scientists

**DOI:** 10.20411/pai.v11i1.970

**Published:** 2026-05-31

**Authors:** Simone Tunesi, Graham Bothamley, Gunar Günther, Yousra Kherabi, Liga Kuksa, Berit Lange, Christoph Lange, Natalie Lorent, Francesca Saluzzo, Martina Sester, Xenia Emilie Sinding Iversen, Marc Tebruegge, Conor Tweed, Anca Vasiliu, Lorenzo Guglielmetti, for the Tuberculosis Network European Trials group (TBnet)

**Affiliations:** 1 Infectious Diseases Unit, AOU SS Antonio e Biagio e C. Arrigo, Alessandria, Italy; 2 Department of Public Health and Pediatric Sciences, University of Turin, Italy; 3 Homerton University Hospital, London, United Kingdom; 4 Queen Mary University of London, London, United Kingdom; 5 London School of Hygiene and Tropical Medicine, London, United Kingdom; 6 Department of Pulmonology, Allergology and Clinical Immunology, Inselspital Bern, Bern University Hospital, Bern, Switzerland; 7 Department of Clinical Sciences, School of Medicine, University of Namibia, Windhoek, Namibia; 8 Infectious and Tropical Diseases Department, Bichat-Claude Bernard Hospital, Assistance Publique-Hôpitaux de Paris, Université Paris Cité, Paris, France; 9 Université Paris Cité, Inserm, IAME, Paris, France; 10 Tuberculosis and Lung Disease clinic, Riga East University hospital, Riga, Latvia; 11 Department of Epidemiology, Helmholtz Centre for Infection Research, Braunschweig, Germany; 12 German Center for Infection Research, TI BBD, Braunschweig, Germany; 13 Infectious Diseases, Research Center Borstel, Leibniz Lung Center, Borstel, Germany; 14 Clinical Tuberculosis Unit, German Center for Infection Research (DZIF), Hamburg-Lübeck-Borstel-Riems, Germany; 15 Respiratory Medicine and International Health, University of Lübeck, Germany; 16 Baylor College of Medicine and Texas Children´s Hospital, Global TB Program, Houston, Texas; 17 Department of Respiratory Diseases, University Hospital Leuven, Leuven, Belgium; 18 Department of Chronic Diseases, Metabolism and Aging, Laboratory of Respiratory Diseases and Thoracic Surgery (BREATHE), KU Leuven, Leuven, Belgium; 19 IRCCS San Raffaele Scientific Institute, Milan, Italy; 20 Vita salute San Raffaele University, Milan, Italy; 21 Department of Transplant and Infection Immunology, Saarland University, Homburg, Germany; 22 Centre for Gender-specific Biology and Medicine (CGBM), Saarland University, Homburg, Germany; 23 International Reference Laboratory of Mycobacteriology, Statens Serum Institut, Copenhagen, Denmark; 24 Department of Child and Adolescent Medicine & Austrian National Reference Centre for Childhood Tuberculosis, Klinik Ottakring, Vienna Healthcare Group, Vienna, Austria; 25 Infectious Diseases Network, Vienna Healthcare Group, Vienna, Austria; 26 Department of Pediatrics, University of Melbourne, Parkville, Australia; 27 MRC Clinical Trials Unit, University College London, London, United Kingdom; 28 Department of Infectious, Tropical Diseases and Microbiology, IRCCS Sacro Cuore Don Calabria Hospital, Negrar di Valpolicella, Verona, Italy

**Keywords:** NTM, TB, TBnet, pediatric TB

## Abstract

**Background:**

Tuberculosis (TB) remains a major cause of morbidity and mortality worldwide, while infections from non-tuberculous mycobacteria (NTM) represent a growing public health threat. In its 20^th^ year of activity, the Tuberculosis Network European Trials group (TBnet; www.tbnet.eu) is one of the leading research networks in Europe. In this review, we summarize the main advances in TB and NTM that occurred in 2025.

**Methods:**

We conducted a non-systematic review of articles published in 2025, with a strong emphasis on research with current or potential impact on clinical practice. We also selected the most impactful papers regarding the management of adult TB, pediatric TB, and NTM infections.

**Results:**

Members of the TBnet Steering Committee summarize the main advances in their areas of interest, including epidemiology, pathogenesis, prevention, diagnosis, treatment, pediatric TB, and NTM infections. We also include a summary of future research priorities.

**Conclusions:**

The year 2025 presented many exciting advancements in almost all fields of mycobacterial science. This article provides an expert-based, clinically orientated summary of the main new findings in the TB and NTM fields and aims to provide an updated overview of the state of the art in those areas.

## INTRODUCTION

Tuberculosis (TB) still ranks as the leading cause of death from a single infectious pathogen, while other mycobacterial diseases are recognized as increasingly important. Recent World Health Organization (WHO) estimates indicate that the global number of people affected by TB remains at historically high levels, while the epidemiology of the disease shows pronounced global disparities [1]. At the same time, the spectrum of mycobacterial infections is undergoing a notable shift in countries with low TB incidence, where non-tuberculous mycobacteria (NTM) have emerged as an increasingly important cause of pulmonary and extrapulmonary disease. Despite chronically insufficient research and development resources, in addition to relevant funding cuts in the last year, significant progress has been made in the development of novel tools and interventions to tackle mycobacterial infections. These include advanced diagnostic, prevention, and treatment strategies, including new antimycobacterial agents currently undergoing clinical evaluation. This review integrates key findings from studies published in 2025, providing a comprehensive overview of advances in epidemiology, pathogenesis, prevention, diagnosis, and treatment of TB and NTM disease, including post-treatment sequelae, in both adult and pediatric populations.

## EPIDEMIOLOGY

**Figure 1.**
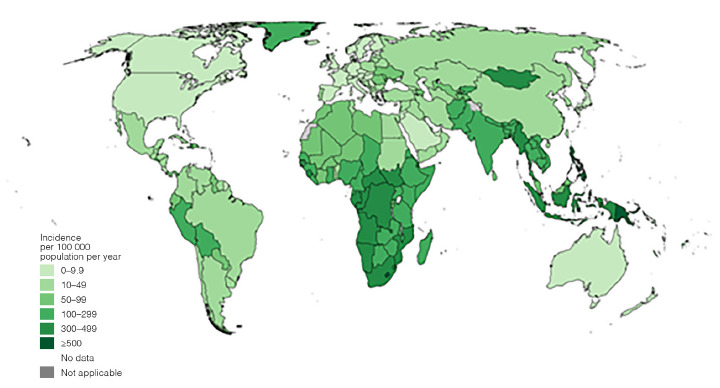
**Estimated TB incidence rates, 2024 (WHO Global TB Report 2025, permission obtained to publish).** TB incidence rates remain alarmingly high in low income countries in sub-Saharan Africa and south-east Asia.

In 2024, an estimated 10.7 million people developed TB and 1.23 million died from the disease worldwide (Figure 1) [1]. This corresponds to a global incidence rate of 131 per 100,000 population and a case fatality rate of 11.5%. TB mortality declined for the third consecutive year, by 29% compared with 2015. Among all people globally who developed TB in 2024, 5.8% were living with HIV, continuing a long-term downward trend from a peak observed in 2000 [1].

Although many indicators have rebounded following the disruptions caused by the COVID-19 pandemic, overall progress remains insufficient [2]. The 12% reduction in TB incidence between 2015 and 2024 falls far short of the End TB Strategy milestone of a 50% reduction by 2025. The importance of this missed step is huge, considering that this checkpoint has been completely missed [3]. This is further complicated by the expected impacts of funding disruptions on initiatives for TB programs. The actual effect of these funding cuts is not yet clear; however, current projections assume that the additional burden of disease might lead to more than 0.5 million additional TB deaths, even if not accounting for cascading effects by disruption to antiretroviral therapy (ART) [4].

The burden of disease remains highly concentrated geographically. Thirty high-TB-burden countries accounted for 87% of all incident cases, and 8 countries—India, Indonesia, the Philippines, China, Pakistan, Nigeria, the Democratic Republic of the Congo, and Bangladesh—accounted for 67% of the global burden (Figure 1) [1]. Regionally, the largest proportions of incident cases occurred in the WHO South-East Asia (34%), the Western Pacific (27%), and Africa (25%). Trends varied substantially by region: between 2015 and 2024, the WHO European Region achieved a 39% reduction in incidence, and the African Region achieved a 28% reduction, with both regions surpassing the first End TB Strategy milestone [5]. TB affects all age groups, but incidence worldwide is highest among adult men (≥15 years), who accounted for 54% of cases, compared with 35% among adult women and 11% among children in 2024.

Drug-resistant TB (DR-TB), particularly multidrug-resistant or rifampicin-resistant TB (MDR/RR-TB), continues to pose a major public health challenge. In 2024, an estimated 390,000 people developed MDR/RR-TB. India, China, the Philippines, and the Russian Federation together accounted for more than half of all estimated MDR/RR-TB cases globally [6]. Emerging resistance to Group A WHO medicines (fluoroquinolones, bedaquiline, linezolid), observed in 2025 in Moldova and Ukraine, will likely challenge efforts to control drug-resistant tuberculosis in Europe a WHO high-burden country of MDR/RR-TB.\nMETHODS: A retrospective cohort study analysed national tuberculosis surveillance data (2021-2022) [7, 8].

## COMORBIDITIES

In 2025, evidence reaffirmed that both communicable and non-communicable comorbidities continue to influence the global burden and clinical course of TB. Recent evidence highlights their persistent epidemiological impact and describes emerging approaches, such as integrated care pathways and predictive tools, to enable earlier diagnosis and coordinated management.

Overall, comorbidities remain insufficiently addressed by national TB programs. A review of high-burden country guidelines shows that recommendations for systematic screening, referral, and coordinated management are often missing [9]. Most guidelines still follow a vertical, single-disease framework that does not reflect the multi-morbidity encountered in practice, limiting early detection and continuity of care. Guideline developers and National TB Program leadership should consider how they might provide more specificity around comorbidity and risk factor screening, diagnosis, and treatment in subsequent iterations of country guidelines [9]. A clinical review reinforces how a broad range of comorbidities, from HIV and diabetes to chronic lung diseases, silicosis, malignancy, and other immunosuppressive states, shape TB presentation and outcomes. These conditions alter immune responses, contribute to atypical clinical features and delays in diagnosis, and are associated with more severe disease and poorer treatment results [10].

### Malnutrition

Malnutrition negatively affects TB outcomes worldwide. A recent study sought to detail the role of body mass index (BMI), confirming that a low BMI is strongly correlated with mortality among patients with TB [11]. An interesting new approach describes how climate change is driving the increase in food prices in low-resource countries, consequently reducing access to food in high TB-prevalence settings [12]. Standardization of the application of nutritional scores in standard clinical management remains an unmet need [13].

### Silicosis

Silicosis and occupational silica exposure are strong risk factors for TB, with studies consistently showing a 2- to 4-fold higher risk of active TB among individuals with silicosis compared with unexposed populations. Silica particles impair alveolar macrophages, disrupt dendritic cell activity, and induce chronic fibrotic and immunomodulatory changes. Elevated TB incidence is also reported among individuals with substantial silica exposure but no diagnosed silicosis, with risk further increased in settings where HIV prevalence is high. The burden is concentrated in industries such as mining, quarrying, construction, and foundry work, underscoring the need for occupational health surveillance, routine TB screening, and dust-control measures in high-risk workplaces [14].

### HIV

HIV remains a major comorbidity influencing TB epidemiology and outcomes, accounting for approximately 6% to 8% of global TB cases and a substantially higher proportion of TB-related mortality. Recent publications describe declines in TB incidence among people receiving timely HIV diagnosis and antiretroviral therapy, yet TB continues to cause substantial mortality. Data from different regions show marked heterogeneity in TB–HIV coinfection patterns. Common challenges include late presentation, the limited sensitivity of smear microscopy in immunosuppressed individuals, and gaps in service integration, particularly for adolescents, older adults, and populations with poor access to care. These findings support efforts to expand rapid testing, improve coordination between HIV and TB services, and promote earlier initiation of ART and TB treatment [15, 16].

### Diabetes Mellitus

Diabetes impacts both TB risk and outcomes, with meta-analyses indicating a 2- to 3-fold increased risk of developing active TB among individuals with diabetes compared to those without. Experimental and clinical evidence show that chronic hyperglycemia impairs immune function, including macrophage activity, cytokine responses, and immune-metabolic pathways, leading to higher mycobacterial burden and slower treatment responses [17]. Genetic analyses using Mendelian randomization strengthen the likelihood of a causal link between diabetes and TB and support the need for bidirectional screening in high-burden settings [18]. Advances in predictive modeling also show potential for identifying individuals at early risk of diabetes–TB comorbidity, enabling more targeted prevention and clinical management [19].

### Other Comorbidities

TB and cancer increasingly intersect clinically and epidemiologically. Chronic TB-related inflammation and fibrosis may contribute to carcinogenesis, while cancer-associated immune suppression can facilitate the persistence or reactivation of *Mycobacterium tuberculosis* (MTB). Diagnostic overlaps are common, and individuals with cancer, particularly those with lung or hematologic malignancies, have 2- to 6-fold higher rates of active and fatal TB. These findings emphasize the need for integrated diagnostic pathways within oncology care [20, 21].

Mental health is also gaining attention. Depression is highly prevalent among individuals with TB, affecting approximately 30% to 50% of patients in many settings, yet it remains under-detected and is associated with poorer treatment adherence and recovery. A co-design approach in a low-resource context demonstrated that incorporating depression screening and basic psychosocial support into TB services delivered by non-specialist staff, is feasible and acceptable [22].

## PATHOGENESIS AND PREVENTION

### Pathogenesis

Among the 4 main human-adapted MTB complex lineages, lineage 1 is increasingly recognized as a strain with lower virulence but prolonged infectiousness [23]. Its frequency in several high-burden countries, such as the Philippines and India, makes this lineage the most common in people affected by TB [24]. In general, variabilities in strains may affect drug resistance, diagnostics, and transmissibility. Reduced expression of RD1 antigens affects virulence and makes contact tracing more difficult. In addition, strains with increased sulfolipid metabolism seem to stimulate cough, which may make transmission more likely [25].

### Prevention by Identification of TB Infection

Active case finding in contact tracing is key to prevention, but is complicated by a high rate of asymptomatic cases [26]. A recent study confirmed that more than 80% of prevalent cases among household contacts were asymptomatic, and chest X-rays missed >40% of those cases. Thus, symptom-based screening in combination with chest X-rays achieved a sensitivity of only 64% in identifying prevalent cases [27]. In a large population-based study in China, elderly individuals with abnormal chest X-rays were found to have a higher risk of developing TB within 2 years, highlighting that chest X-ray abnormalities may identify a high-risk group [28]. When chest X-ray was used in combination with Xpert MTB/RIF Ultra as an initial screening test through event-based and door-to-door screening in Uganda, even trace-positive sputum results enabled prediction of TB disease during 2 years of follow-up, especially when combined with atypical chest X-rays [29]. In 2 cohorts from Brazil and India, an immune signature of interleukin (IL)-8, IL-10, and C-C motif chemokine ligand 3 (CCL3) reliably predicted progression to active TB among QuantiFERON-positive close contacts with a sensitivity and specificity ≥80% [30]. Several single-gene blood RNA transcripts allowed detection of subclinical TB with accuracy comparable to multi-gene signatures, suggesting the possibility of simplified biomarker approaches [31]. A prospective TBnet European multicenter study performed among 5 groups of immunocompromised individuals found that the QuantiFERON-TB Gold Plus assay had limited predictive value for progression to active TB, except among people living with HIV with uncontrolled viral replication and low CD4 T-cell counts. The study emphasizes the need for risk-stratified strategies in low-incidence settings [32]. Another TBnet study specifically addressing progression in transplant recipients found that the highest incidence occurred in patients with positive TB skin test (TST)/interferon-gamma release assay (IGRA) screening who did not receive tuberculosis preventive treatment (TPT). TB cases mainly occurred >2 years after transplantation, which suggests an important role of *de novo* infection in transplant recipients in Europe [33]. Finally, the long-held dogma to delay IGRA testing after live-attenuated vaccination was challenged by data showing that live-virus vaccines (MMR, polio, or varicella) do not cause false-negative TST/IGRA results. This suggests that immediate testing is reliable, could reduce delays in risk assessment, and enable timely TB prevention measures [34].

### Prevention by Vaccination

TB vaccine trials that use disease as the primary outcome are challenging, as progression to active TB is relatively rare. To overcome this, sustained IGRA conversion is increasingly being explored as a surrogate endpoint. Evidence of success from Bacille Calmette-Guérin (BCG) vaccination has been mixed. The efficacy of BCG revaccination to protect against TB infection was tested among HIV-negative adolescents in a phase 2b, double-blind, randomized, placebo-controlled trial that did not show any benefit compared to placebo [35]. A recent phase 2b trial tested the H56:IC31 vaccine for prevention of recurrence or reinfection in HIV-negative adults after more than 5 months of successful treatment for drug-susceptible pulmonary TB. This study even raised concern about possibly inducing relapse, as vaccinated individuals had a slightly higher risk of recurrence as compared to those treated with placebo [36]. In contrast, the M72/AS01E–4 vaccine, which reduced progression to pulmonary TB by about 50% in individuals with TB infection in an earlier phase 2b study, also showed promise in people living with HIV [37], which led to inclusion of people living with HIV in the ongoing phase 3 trial (NCT06062238).

## DIAGNOSTICS

**Figure 2.**
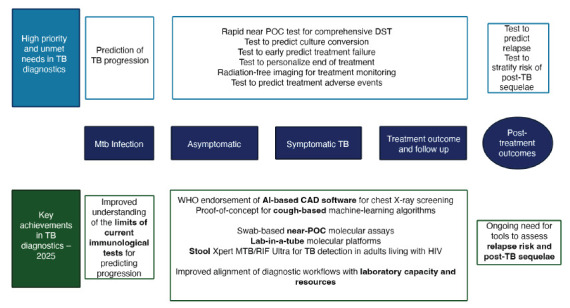
**Early TB diagnosis is a strong priority for WHO and major public health authorities worldwide.** AI-based technologies are playing a key role in the very next future. Adapted from: Lange C, et al. [47]

TB diagnostics are undergoing rapid innovation, with 2025 marking several key advances. Progress has been driven by the dual priorities of simplifying specimen collection and integrating molecular and genomic technologies within streamlined diagnostic algorithms (Figure 2).

### Simplified and Decentralized Molecular Testing

The simplification of specimen collection remains a key goal. Swab-based near-point-of-care (POC) molecular assays such as Truenat MTB Ultima (MTB Ultima) and MiniDock MTB Test (MiniDock MTB) have demonstrated diagnostic performance comparable to standard sputum testing in adults [38]. These assays, which can use tongue or sputum swabs, reduce biosafety risks and enable testing in decentralized settings. Another interesting innovation is the lab-in-a-tube assay, a portable, low-cost molecular platform that integrates sample preparation, amplification, and detection within a single, closed device [39]. Among other non-sputum-based tools, stool testing with Xpert MTB/RIF Ultra has shown potential for TB detection among adults living with HIV, addressing a recognized diagnostic gap in this population [40]. Together, these studies indicate that the diagnostic landscape is moving toward less invasive, more adaptable sampling strategies.

New evidence finally suggests that urinary and sputum LAM could be a useful diagnostic tool, fitting WHO target product profile for the diagnosis of TB, even in HIV-negative patients [41, 42].

### Integration into Diagnostic Algorithms

Since the WHO endorsed targeted next-generation sequencing (tNGS) for drug-resistant TB diagnosis in 2024, attention in 2025 has shifted to its implementation and integration into diagnostic algorithms [43]. Ongoing work focuses on bacterial-load thresholds, workflow adaptation, and algorithm placement. At the systems level, the European Reference Laboratory Network for Tuberculosis (ERLTB-Net) has outlined a new algorithmic framework for the EU/EEA incorporating rapid molecular assays, sequencing, and selective phenotypic confirmation [44] (Figure 2).

### Advances in Non-Microbiological Screening Tools

Several advances have occurred in non-microbiological diagnostics. The 2025 WHO policy statement on computer-aided detection (CAD) software endorsed 6 tools for the artificial-intelligence (AI)-based interpretation of chest X-rays. This follows growing evidence that automated imaging analysis can standardize screening quality and increase access to diagnosis in high-burden, resource-limited settings [45].

Complementary progress has been reported in the development of cough-based diagnostic algorithms. Results from the CODA TB DREAM Challenge demonstrated that machine-learning models trained on cough acoustics and minimal clinical data could differentiate pulmonary TB from other respiratory conditions with high accuracy [46]. These findings indicate the potential of digital phenotyping for large-scale, non-invasive screening, especially where access to radiography or microbiological testing remains limited.

### Diagnostic Challenges in Immunocompromised Hosts

Despite these advances, diagnostic gaps remain among immunocompromised individuals. The aforementioned TBnet study reiterated that the QuantiFERON-TB Gold Plus assay has limited predictive value for progression to active disease [32], underscoring the need for more specific biomarkers that can distinguish TB infection from disease [47].

## TREATMENT

The year 2025 saw eagerly awaited results for phase 2 and phase 3 trials in the treatment of TB disease. Novel agents continue to show promise in early-phase studies; additional results from studies on treatment shortening and treatment intensification were released; and some advances were seen in the management of rifampin- and fluoroquinolone-resistant disease. At the time of this review, there are also multiple studies underway and planned to investigate the treatment effect of host-directed therapies in TB disease, which is an important emerging focus in TB research, but there were no published results for 2025.

### New Drugs Assessed in Early-Phase Trials

**Figure 3.**
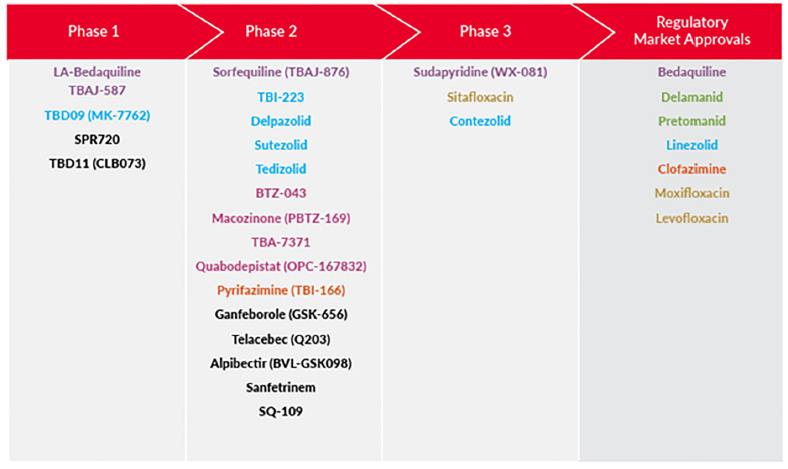
**Global New TB Drug Pipeline showing new compounds being developed for the treatment of tuberculosis** (courtesy of Tejaswini Dharmapuri Vachaspathi et al., Treatment Action Group 2025 - TB Treatment Pipeline Report). Host-directed interventions are not included.

Figure 3 illustrates the pipeline of novel TB drugs, and identifying new oxazolidinones with improved safety profiles is among the main priorities in clinical research. The phase 2B PanACEA-DECODE-01 trial investigated the efficacy, safety, and optimal dosing of 16 weeks of delpazolid treatment in combination with bedaquiline (B), delamanid (D), and moxifloxacin (M) for pulmonary tuberculosis [48]. Pharmacokinetic-pharmacodynamic modeling estimated that once-daily 1,200 mg delpazolid resulted in 38% (95% CI 4-83%) faster decline in time-to-positivity (TTP) than without delpazolid. No significant oxazolidinone-class toxicities occurred except for one serious adverse event of anemia with delpazolid 800 mg twice daily. Delpazolid is planned for inclusion in Wave II of phase 2B in the PARADIGM4TB trial.

### Treatment-shortening and Treatment-intensifying in Drug-susceptible Disease

In 2023, the TRUNCATE-TB trial demonstrated that an 8-week regimen containing bedaquiline-linezolid was non-inferior to standard treatment for drug-susceptible disease when part of a strategy of treatment followed by intensive monitoring and re-treatment as necessary [49]. In 2025, an analysis of the safety and efficacy of the 8-week treatment arms in TRUNCATE-TB was published [50]. With the possibility of re-treatment excluded, 7 of 181 (4%) participants had an unfavorable outcome with standard care, compared to 46 of 184 (25%) in the high-dose rifampicin-linezolid arm and 26 of 189 (14%) in the bedaquiline-linezolid arm. These findings call into question the role of the bedaquiline-linezolid arm in settings where intensive monitoring is not feasible.

The phase 2C Clo-Fast study randomized participants to either a 3-month regimen of rifapentine-isoniazid-ethambutol-clofazimine (with pyrazinamide for the first 8 weeks) or standard of care for drug-susceptible disease [51]. Despite similar 12-week culture conversion rates in the experimental and control arms, respectively, the probability of an unfavorable outcome at week 65 was 52% (95% CI 37-69) and 27% (95% CI 14-50), and the trial was stopped early due to lack of clinical efficacy. The safety profile was undesirable in the clofazimine-containing arm, with 45% experiencing a grade 3 or higher adverse event compared to 16% in the control arm.

The HARVEST trial was a randomized, blinded, placebo-controlled trial of oral first-line TB treatment for TB meningitis with high-dose rifampin (35 mg/kg) for the first 8 weeks compared to standard therapy with 10 mg/kg rifampin throughout a 9- to 12-month treatment course [52]. Of 499 participants, 60.9% were HIV-infected. There was no significant difference in the primary outcome of mortality at 6 months post-randomization between the intervention and control groups, and no significant differences across subgroups. Treatment-emergent adverse events were reported by approximately 50% of participants on both arms, with 8.0% experiencing a grade 3 or 4 drug-induced liver injury in the experimental arm and 4.4% in the control arm. Overall, the potential for a harmful effect could not be ruled out.

### New Regimens for Drug-resistant Tuberculosis

The endTB study investigated the efficacy of five 9-month oral regimens containing bedaquiline, delamanid, linezolid (L), levofloxacin (Lfx) or moxifloxacin, clofazimine (C), and pyrazinamide (Z) in rifampicin-resistant, fluoroquinolone-susceptible disease [53]. Non-inferiority, based on a 12 percentage points margin, was demonstrated by 3 experimental arms compared to the WHO-recommended standard therapy in both populations for the primary analysis (BCLLfxZ, BLMZ, and BDLLfxZ). Safety profiles were comparable across the non-inferior experimental arms and standard of care, except for increased hepatotoxicity in the BCLLfxZ and BLMZ arms. This study further expanded the armamentarium for rifampin-resistant TB. There is now growing pressure to ensure access to these life-saving drugs globally.

In parallel, the endTB-Q study randomized participants with rifampicin- and fluoroquinolone-resistant TB to receive either bedaquiline-delamanid-linezolid-clofazimine (BDLC) for 39 weeks or 24 weeks (based on disease severity and treatment response) [54] or the WHO-recommended longer regimen. BDLC was not non-inferior in the modified intention-to-treat but not in the per-protocol analysis. The authors conclude that a longer treatment course may be required in severe disease with rifampicin- and fluoroquinolone-resistance. These results also highlight the importance of ongoing work to clearly and reproducibly define disease severity for both clinical research and practice.

These exciting new developments are counterbalanced by sobering outcomes of treatment in people affected by bedaquiline-resistant and extensively drug-resistant TB (XDR-TB), where treatment success rates are similar to those of the pre-antibiotic era [55, 56].

## POST-TB DISEASE

The first definition of post-tuberculosis lung disease (PTLD) stems from 2019, describing the condition as “Evidence of chronic respiratory abnormality, with or without symptoms, attributable at least in part to previous tuberculosis.” [57]. A more precise, but still broad research definition was published in 2025, including (a) a history of pulmonary or pleural TB without signs of active disease, (b) abnormalities in at least two out of three domains (lung function, imaging and respiratory symptoms) and (c) attributability of radiological, functional or symptomatic impairment at least in part to previous TB [58]. The authors stress the fact that very limited evidence was available to create this definition, but together with the reporting framework, it should be a basis for harmonizing PTLD research.

Among the attempts to gain more insight into the pathogenesis of PTLD, 2 studies should be highlighted. An analysis of immune, metabolic, anatomical and functional features of PTLD within 6 months post-TB treatment documents the complexity and variability of the condition. While inflammation in PET-CT correlated well with lung function impairment, it did not correlate with exercise tolerance, symptoms, and quality of life [59]. The study was conducted in human lungs post-TB and employed single-cell sequencing, demonstrating gene expression patterns associated with senescence, inflammation, fibrosis, and apoptosis. Further, the study demonstrated low levels of FOXO3, a protective longevity gene and NF-κB-driven thrombo-inflammatory post-TB vascular injury [60].

The proceedings from the Third International Post-Tuberculosis Symposium 2025 summarize the current state of discussion and knowledge around PTLD and other TB sequelae, based on input from different working groups covering the following topics: patient engagement; epidemiology and modelling; pathogenesis; post TB lung disease; cardiovascular and pulmonary vascular; central nervous system and musculoskeletal; pediatrics; economic, social and psychosocial aspects; advocacy, policy and stakeholder engagement [61].

To date, existing data on PTLD in children remain very limited, as highlighted by a recent systematic review. The authors found that approximately 40% of children aged 5 to 10 years exhibited abnormal lung function after TB treatment, rising to 65% in patients aged >10 years. Further data are urgently needed to generate a more detailed picture of associated growth and developmental issues [62].

## PEDIATRICS

Recent TB incidence figures in children and adolescents in the EU/EEA give cause for concern. A study using data from 2015-2023 from TESSy at ECDC, found that following a nadir of 1,142 cases in 2021, numbers rose for 2 consecutive years to 1,689 cases in 2023, which was an astonishing increase of 47.9% [63]. Only a minority (28.7%) of cases were microbiologically confirmed. Several factors may have influenced this increase, including improved diagnosis and reporting of pediatric TB, modifications in population movements, and the impact of the COVID-19 pandemic on healthcare services.

In a study from South Africa, 154 (35.8%) of 430 TB-exposed children had TB, and 55 (35.7%) cases were asymptomatic. Unexpectedly, of the children with confirmed TB overall, 17 of 21 (81.0%) were asymptomatic, and only 4 of 21 (19.0%) were symptomatic. The authors concluded that asymptomatic children with TB need to be considered in future treatment-decision algorithms [64].

A multicenter study by the Pediatric TB Network European Trials Group (ptbnet) that focused on congenital (cTB) and postnatal (pTB) TB in the European setting found that cTB was associated with premature birth and low birth weight [65]. Most patients were microbiologically confirmed, but immune-based tests (TST and IGRAs) had poor sensitivity in both groups. Fatal outcome and long-term sequelae were observed in a substantial proportion of patients (8.7%, each), despite the high-resource setting.

A large multicenter study from the Spanish Pediatric TB Research Network (pTBred), which included children with TB in Spain and Mozambique, showed that chest X-rays, even when interpreted by expert readers, have limited sensitivity for detecting TB. Interestingly, lateral X-rays increased the overall sensitivity substantially [66].

A secondary analysis of data from the RaPaed-TB study illustrated that molecular testing cannot yet fully replace mycobacterial cultures [67]. Of 239 children with microbiologically confirmed TB, 110 (46%) were detected by both Xpert MTB/RIF Ultra and culture, 86 (36%) by Xpert MTB/RIF Ultra alone, and 43 (18%) by culture alone. The study also found that analyzing more than one clinical sample increases the diagnostic yield substantially (from 20% to 29%).

Microbiological confirmation of TB disease in children remains challenging, partly due to the inability of young children to generate sputum samples. Several studies have investigated alternative clinical samples that are easier to obtain, including oral swabs, nasopharyngeal aspirates (NPAs), and stool (68–74) by gastric aspiration or sputum induction, has a low diagnostic yield. In this study, we aimed to evaluate the diagnostic performance and additive yield of a novel stool-based assay in children diagnosed with tuberculosis in sub-Saharan Africa.\nMETHODS: We conducted a prospective case-control study from October 2020 to June 2023 in Eswatini, Mozambique, and Tanzania. Children under 15 years newly diagnosed with tuberculosis completed clinical examination, chest radiography, culture, sputum Xpert Ultra, stool Xpert Ultra, and stool-based quantitative polymerase chain reaction (stool qPCR [68–74].

A study in children aged <5 years, using 2 oral swabs analyzed with Xpert MTB/RIF Ultra assays, found that the sensitivity of this approach was only 6.9% against a microbiological reference standard and 1.8% against a composite reference standard [66]. A large multi-country study in children aged <15 years evaluating NPAs and stool reported that both sample types had suboptimal sensitivity [73]. Two recent studies investigated whether improvements in stool processing can increase the diagnostic yield. One assessed 3 centrifuge-free processing methods, comprising a simple one-step method, a stool processing kit, and an optimized sucrose flotation method, and found that each achieved similar sensitivity [74]. Another study in children in India, South Africa, and Uganda confirmed these findings [69]. Taken together, current evidence shows that, in children, respiratory samples (ie, sputum or gastric aspirates) remain superior to samples that are more easily obtained.

In parallel, the search for better biomarkers continues. A study investigated plasma metabolic signatures in children with TB, identifying a nine-metabolite signature that achieved an area under the curve (AUC) value of 0.72 for distinguishing confirmed TB from unlikely TB [75]. Another study used high-throughput proteomics, which identified 4 biosignatures (comprising 3-6 proteins) AUCs of 0.87-0.88 [76]. A third study used a CRISPR-based approach to detect MTB DNA in blood from children with TB; although the sample size was small (n=27), the results indicated that the assay used had greater sensitivity than Xpert MTB/RIF assays performed on respiratory samples from the same patients [39]. Those studies are encouraging, but their findings require further validation in independent cohorts, especially considering that previous proteomic studies in adults have found different sets of proteins to be characteristic markers of TB [77, 78].

A systematic review, which included data from ptbnet, provided insights regarding the treatment and outcomes of children and adolescents with rifampicin- and multidrug-resistant TB. The findings were sobering – it showed that, out of 23,369 children and adolescents with rifampicin- and multidrug-resistant TB, only 72.0% had treatment success, while 12.2% died, 3.1% experienced treatment failure, and 12.7% were lost to follow-up [79]. Table 1 summarizes the most important new evidence in Pediatric TB.

**Table 1.** Highlighted 2025 Publications in Pediatric TB According to TBnet Steering Committee Members

Article title	Impact
Mulenga et al. **Asymptomatic tuberculosis in children with household exposure to Mycobacterium tuberculosis.** *Clin Infect Dis*. 2025. [64]	This study showed that a substantial proportion (35.7%) of children diagnosed with TB disease during household contact investigations had asymptomatic (or subclinical) TB.
Götzinger et al. **Clinical presentation, diagnostics, and outcomes of infants with congenital and postnatal tuberculosis: a multicentre cohort study of the Paediatric Tuberculosis Network European Trials Group (ptbnet).** *Lancet Reg Health Eur*. 2025 [65]	Multicenter study conducted by ptbnet that represents an important addition to the limited literature on congenital and perinatal TB, which are rare in Europe, but associated with substantial morbidity and mortality.
Olbrich et al. **Sequential and parallel testing for microbiological confirmation of tuberculosis disease in children in five low-income and middle-income countries: a secondary analysis of the RaPaed-TB study.** *Lancet Infect Dis*. 2025 [67]	Important secondary analysis of data from the RaPaed-TB study, which showed that analysis of two or more clinical samples increases the diagnostic yield in children substantially. The study findings also underscore the importance of using culture- and PCR-based methods in parallel.
Hernanz-Lobo et al. **Diagnostic performance of chest radiography for pediatric tuberculosis across high- and low-burden settings.** *Front Pediatr*. 2025. [80]	A multicenter study in children with TB disease in Spain and Mozambique, that confirmed that chest X-rays, even when read by experts, have low sensitivity in detecting TB if used in isolation.
Garcia-Prats et al. **Characteristics of children and adolescents with multidrug-resistant and rifampicin-resistant tuberculosis and their association with treatment outcomes: a systematic review and individual participant data meta-analysis.** *Lancet Child Adolesc Health.* 2025 [79]	A comprehensive review and detailed individual patient data meta-analysis (with data from 42 studies), offering insights into the treatment and outcome of children and adolescents with drug-resistant TB. A key finding was the association between the use of Group A anti-TB drugs (bedaquiline, levofloxacin, moxifloxacin and linezolid) and improved treatment outcomes.

## NONTUBERCULOUS MYCOBACTERIAL DISEASE

In 2025, epidemiologic and translational advances continued to shape understanding of nontuberculous mycobacterial (NTM) disease (Table 2). A Danish registry study of all NTM isolates from 1991–2022 analyzed 4,123 first-time positive individuals and observed increasing pulmonary NTM isolation rates, particularly among older females: in 2008–2022 vs 1991–2007, the incidence rate ratio for females was 1.9 (95% CI, 1.7–2.1) and for males 1.3 (95% CI, 1.1–1.4) (81). Geographic heterogeneity was evident, with age- and sex–adjusted rates 10% to 40% higher in rural and provincial municipalities vs the capital region [81].

**Table 2.** Highlighted 2025 Publications in NTM Disease According to TBnet Steering Committee Members

Article title	Impact
Dousa et al. **The Role of β-Lactam Antibiotics in Treating Mycobacterium abscessus: From Laboratory Insights to Clinical Applications and the Case for Clinical Trials.** *Clin Infect Dis*. 2025 [88]	This comprehensive review summarizes laboratory insights and clinical experience of beta-lactam/beta-lactamase inhibitor-based treatment regimens for *M. abscessus* infections.
Sulaiman et al. **Optimising Non-Pharmacological Interventions in People with Non-Tuberculous Mycobacterial Pulmonary Disease: A Systematic Review.** *ERJ Open Research.* 2025 [94]	This paper addresses the potential of non-pharmacological interventions for NTM-PD yet highlighting the need for high-quality longitudinal studies and RCTs to establish the efficacy of interventions such as airway clearance, pulmonary rehabilitation and nutritional support.
Dartois et al. **Next-generation rifamycins for the treatment of mycobacterial infections.** *Proc Natl Acad Sci U S A*. 2025 [85]	This paper reports the development of orally active, next-generation rifamycin analogs that overcome intrinsic resistance and drug-drug interactions, showing enhanced bactericidal potency in preclinical models, offering a potentially promising new drug class to improve *M. abscessus* treatment outcomes
Shrivastava et al. **Sulbactam-durlobactam improves cephalosporin and carbapenem susceptibility and time-kill effect against Mycobacterium abscessus.** *Microbiol Spectr.* 2025 [95]	This study shows the in vitro potential of sulbactam-durlobactam (Sul/Dur) to restore the activity of several beta-lactams against *M. abscessus* and propose to advance the combination of Sul/Dur with imipenem and ceftriaxone as a double beta-lactam beta-lactamase backbone regimen in *M. abscessus*-PD.
Trinh et al. **Understanding recurrence in Mycobacterium avium complex pulmonary disease: genotypic strategies to support clinical decision-making.** *J Clin Microbiol.* 2025 [96]	This study provides an insight into the role of molecular diagnostics in the understanding of recurrence mechanisms in MAC-PD and proposes their integration into routine care to support clinical decision-making.

Environmental risk associations were reinforced. In U.S. cohorts, higher molybdenum and vanadium concentrations in drinking water, and chloramine disinfectant use have been correlated with increased pulmonary NTM, with effects varying by geographic region [82].

Persons with underlying lung disease and suppressed immunity remain particularly vulnerable to NTM infection [83]. In a multinational matched case–control study of 85 solid organ transplant recipients diagnosed with NTM disease (2008–2018), the median time to diagnosis was 1.8 years post-transplant; 34% were lung recipients. One-year mortality was 20%, higher than matched uninfected controls, and lack of NTM therapy worsened outcomes [84]. These findings emphasize the need for surveillance in high-risk populations and for effective, well-tolerated regimens.

Preclinical drug development for *M. abscessus* showed notable advances. In an engineered C25-modified model, rifabutin analogs (UMN-120, UMN-121) have been shown to evade intrabacterial ADP-ribosylation, avoid CYP3A4 induction, and retain potent bactericidal activity. In murine lung models, these compounds cleared *M. abscessus* as effectively as standard 4-drug therapy, highlighting their potential as oral backbone agents [85]. A new all-oral, bactericidal triple-drug regimen (tebipenem + avibactam, moxifloxacin, and rifabutin) has been presented, which potentiated killing against both replicating and non-replicating, drug-tolerant bacteria, including in surrogate caseum models [86].

Meanwhile, dual β-lactam (or β-lactam/β-lactamase inhibitor) strategies also received renewed attention. *M. abscessus* cell-wall synthesis seems to involve DD and LD transpeptidases, which can be differentially targeted by β-lactam combinations. In vitro synergy was observed with cefoxitin/imipenem, ceftaroline/imipenem, ceftazidime/ceftaroline, and ceftazidime/imipenem, even without β-lactamase inhibitors [87, 88]. Although clinical evidence is limited, case reports support translational potential.

In *M. avium* complex (MAC) pulmonary disease, retrospective East Asian data suggest that dual regimens (macrolide + ethambutol) may be effective in select patients, though resistance and relapse remain a concern [89, 90]. Results from ongoing trials comparing 2- vs 3-drug regimens (NCT03672630, NCT04677569) are pending.

Host-directed strategies, including inhaled nitric oxide, GM-CSF, autophagy modulation, and interferon-gamma-driven macrophage activation, continue to attract interest [91], but no major breakthroughs occurred in 2025. Mechanistic and early-phase translational studies are ongoing [92, 93], though large-scale clinical translation remains aspirational.

## CONCLUSIONS

The field of mycobacterial diseases is undergoing rapid development. The year 2025 has been one of overturning long-held and cherished beliefs, with important benefits for those being treated for TB (Table 3). Higher rifampicin doses have been promoted on the basis of improved bactericidal activity. However, adverse events have been higher than expected, especially when including grades 1 and 2 adverse events [97], with no improvement in treatment outcome of severe TB disease, such as meningitis (50,52,98,99) multi-stage, open-label, randomised controlled trial in which participants aged 18-65 years with rifampicin-susceptible pulmonary tuberculosis were randomly assigned via a web-based system, using permuted blocks, to 24-week standard treatment (rifampicin, isoniazid, pyrazinamide, and ethambutol [50, 52, 98, 99].

**Table 3.** Key Recommendations for Tuberculosis Prevention, Diagnosis, and Treatment by the World Health Organization (WHO) Issued in 2025

Recommendations	Ref.
For **adults and adolescents** with signs or symptoms of TB or who **screened positive for pulmonary TB, low-complexity automated NAATs should** be used on respiratory samples as **initial diagnostic tests for TB,** rather than smear microscopy or culture.	[113]
For people with signs and symptoms of **extrapulmonary TB, low-complexity automated** NAATs on **lymph node tissue aspirate, pleural tissue, pleural fluid, synovial fluid, peritoneal fluid or pericardial fluid should be used** for the initial diagnosis of TB, rather than smear microscopy or culture.	[113]
For **children** who are **HIV-negative or have an unknown HIV status,** who have signs or symptoms or screen positive for **pulmonary TB, concurrent testing using low-complexity automated NAATs on respiratory and stool samples should be used** as the initial diagnostic strategy for diagnosing TB, rather than low-complexity automated NAATs on respiratory or stool samples alone.	[113]
People aged 12 years or older with **drug-susceptible pulmonary TB**, may receive a **4-month regimen of isoniazid, rifapentine, moxifloxacin and pyrazinamide.**	[114]
WHO suggests the use of the **6-month treatment regimen composed of bedaquiline, pretomanid, linezolid (600 mg) and moxifloxacin (BPaLM**) rather than 9-month or longer (18-month) regimens in **MDR/RR-TB** patients.	[114]
WHO suggests the use of a **6-month treatment regimen composed of bedaquiline, delamanid, linezolid (600 mg), levofloxacin, and clofazimine (BDLLfxC) in MDR/RR-TB patients** with or with-out fluoroquinolone resistance.	[114]
WHO suggests the use of the **9-month all-oral regimen** rather than longer (18-month) regimens in patients with MDR/RR-TB and in whom resistance to fluoroquinolones has been excluded.	[114]
WHO suggests using the **9-month all-oral regimens** (**BLMZ, BLLfxCZ and BDLLfxZ**) over currently recommended longer (>18 months) regimens in patients with MDR/RR-TB and in whom resistance to fluoroquinolones has been excluded. Amongst these regimens, using BLMZ is suggested over using BLLfxCZ, and BLLfxCZ is suggested over BDLLfxZ.	[114]
WHO suggests against using **9-month DCLLfxZ or DCMZ regimens** compared with currently recommended longer (>18 months) regimens in patients with fluoroquinolone-susceptible MDR/RR-TB.	[114]
In multidrug- or rifampicin-resistant tuberculosis (MDR/RR-TB) patients on **longer regimens, all three Group A agents and at least one Group B agent** should be included to ensure that **treatment starts with at least four TB agents likely to be effective**, and that at least three agents are included for the rest of the treatment if bedaquiline is stopped. If only one or two Group A agents are used, both Group B agents are to be included. **If the regimen cannot be composed with agents from Groups A and B alone, Group C agents are added to complete it**.	[114]
A **high-sensitivity, high-specificity screening test** that can be implemented as a single screening step before referral for diagnostic testing or effective ruling out of TB should have at **minimum a sensitivity of 90% and specificity of 80%** for the diagnosis of tuberculosis.	[115]
A **package of interventions** including **screening, treatment and/or prophylaxis for major opportunistic infections, rapid ART initiation** and **intensified adherence support** interventions should be offered to everyone presenting with **advanced HIV disease**	[116]
**Household contacts of people with TB** should be **offered nutritional assessment and counselling** as part of contact tracing. If undernutrition is identified, it should be managed according to WHO guidance.	[116]

Biomarkers have been sought to assess the likelihood of an outcome. However, the best indices for relapses, using data gathered from RIFASHORT [100] and TRUNCATE-TB [50], now appear to be Xpert cycle threshold at the start of treatment and the return of symptoms. The latter is especially interesting in view of the lack of sensitivity and specificity of symptoms in TB diagnosis. Overall, these and other markers, such as the radiologic extent of TB disease, may pave the way to promising new approaches of stratified medicine for TB treatment [53].

Never before in the history of TB have there been more anti-tuberculosis compounds in phases 1-3 clinical evaluation. However, current first-line regimens are still based on rifamycin, while MDR/RR-TB regimens include both bedaquiline and linezolid, the latter burdened by substantial toxicity. The increasing burden of bedaquiline-resistant and XDR-TB, for which no shorter and effective regimen exists, is deeply concerning. Pre-approval access to new compounds in late-stage development is a priority to treat patients with bedaquiline-resistant TB [101].

A plethora of novel diagnostic tools is “close-to-clinical application” with the potential to improve TB diagnostics. Promising approaches include the detection of MTB DNA at lower thresholds in serum, extracellular vesicles, and exosomes using CRISPR technology [39], and the rapid detection of T-cell responses in a point-of-care test that may potentially replace the current IGRAs [102]. This new technique holds the promise for broader applicability to understanding the immune response to MTB and translating the single-cell RNA sequencing studies of granulomas into a diagnostic test of clinical value [103–105]. Finally, in the 2025 Global Tuberculosis Report, WHO lists 18 TB vaccines in clinical development, including 6 in phase 3 trials, which will hopefully improve TB prevention compared to the more than 100-year-old BCG vaccine.

Despite these developments, the incidence of TB is still at a historical peak of more than 10 million estimated persons being newly affected in 2024 [1]. Although antituberculosis medicines are on the WHO’s list of essential medicines [106], stock-outs of these medicines are not infrequent, for instance in the Philippines [107] and even in Europe [108]. Rifapentine, a medicine recommended to shorten the duration of TB preventive therapy and TB treatment, is unavailable in almost all EU/EEA countries, as the drug has never been licensed by the European Medicines Agency [109]. The implementation of new, shorter treatments for drug-resistant TB happens with substantial delays in some countries due to problems in the roll-out of pretomanid [110]. Antimicrobial stewardship strategies for TB are urgently needed, as MDR/RR-TB was identified in 2024 as one of 4 antimicrobial-resistant organisms with the highest risk to threaten human health [111] (Table 5).

Decisions on resources made in 2025 are threatening TB control globally. Despite all innovations in research and development, forecasts predict that withdrawal of international donors from funding TB programs may lead to a substantial increase in the global TB incidence and attributed deaths in the next 10 years [112]. The growing field of NTM diseases is still largely ruled by eminence-based medicine, with a large need to fill the knowledge gap in all areas, from prevention to diagnosis and treatment.

In conclusion, we can assume that despite 2025 being a fruitful year across almost all TB research fields (Table 4), many research priorities still need to be addressed by authorities and stakeholders. Rapid, affordable, and cheaper diagnostic tools are mandatory to grant early diagnosis, especially in specific populations such as children or people living in low-resource, high-prevalence countries. TB and especially NTM treatments remain long, but new evidence is adding strength, year after year, to shorter and more tolerable schemes. Patient management evidence, finally, needs to be enforced not only for TB active disease, but also for long-term sequelae and concomitant diseases (Table 5).

**Table 4.** Highlighted 2025 Publications in Adult TB According to TBnet Steering Committee Members

Paper	Comment
Guglielmetti et al. **Oral Regimens for RifampinResistant, FluoroquinoloneSusceptible Tuberculosis.** *NEJM*. 2025. [53]	Phase 3, multinational, open-label, randomized, controlled non-inferiority trial comparing standard therapy for treatment of fluoroquinolone-susceptible, rifampin-resistant tuberculosis with five 9-month oral regimens. In the modified intention-to-treat analysis, 80.7% of the patients in the standard-therapy group had favorable outcomes. Non-inferiority has been confirmed for 3 bedaquilin-based regimens: BCLLfxZ, 9.8 percentage points (95% CI, 0.9 to 18.7); BLMZ, 8.3 percentage points (95% CI, −0.8 to 17.4); BDLLfxZ, 4.6 percentage points (95% CI, −4.9 to 14.1). Grade 3 or higher hepatotoxic events occurred in 11.7% of participants overall and in 7.1% of those receiving standard therapy.
Youngquist et al. **Rapid tuberculosis diagnosis from respiratory or blood samples by a low cost, portable lab-in-tube assay.** *Science Transl Med*. 2025. [39]	Description of a low complexity, lab-in-tube point-of-care system that is read by an integrated handheld device that detects *Mycobacterium tuberculosis* (MTB) DNA in blood and respiratory samples from a variety of clinical settings. This is a d CRISPR-Cas12a reagents-based technology attaining singlenucleotide specificity and high sensitivity within 1 hour of sample application, without a conventional DNA isolation procedure. Assay results obtained with serum cell–free DNA isolated from a cohort of children aged 1 to 16 years detected pulmonary and extrapulmonary TB with high sensitivity vs culture and GeneXpert MTB/RIF results (81% vs 55% and 68%) and good specificity (94%), meeting the WHO target product profile criteria for new non-sputum TB diagnostics.
Guglielmetti et al. **Bedaquiline, delamanid, linezolid, and clofazimine for rifampicin-resistant and fluoroquinolone-resistant tuberculosis (endTB-Q): an open-label, multicentre, stratified, non-inferiority, randomised, controlled, phase 3 trial.** *Lancet Resp Med*. 2025. [54]	Open-label, multicenter, stratified, non-inferiority, randomized, controlled, phase 3 trial. A total amount of 1,030 adult patients affected pulmonary TB with resistance to rifampicin and fluoroquinolones were included. Participants were randomly assigned (2:1) to a 39-week BDLC regimen or to the control group (intended as individualized WHO-recommended longer standard of care). Moreover, in the BDLC group, 29% of patients were assigned to receive the 6-month regimen and 71% the 9-month regimen. Randomization was stratified by country and baseline disease extent. At week 73, favorable outcome was reached by 87% of participants in the BDLC group vs 89% in the control group in the modified intention-to-treat (mITT) population. Overall non-inferiority was not shown and grade 3 or higher adverse events were reported in 68% and 73% of patients, respectively.
Paton et al. **Efficacy and safety of 8-week regimens for the treatment of rifampicin-susceptible pulmonary tuberculosis (TRUNCATE-TB): a prespecified exploratory analysis of a multi-arm, multi-stage, open-label, randomised controlled trial.** *Lancet Infect Dis*. 2025. [50]	Multi-arm, multi-stage, open-label, randomized controlled trial in which adult participants with rifampicin-susceptible pulmonary tuberculosis were randomly assigned to 24-week standard treatment (rifampicin, isoniazid, pyrazinamide, and ethambutol) or the TRUNCATE regimens: Rifampicin-linezolid 8-week regimen (n=184); rifampicin-clofazimine 8-week regimen (n=78); rifapentine-linezolid 8-week regimen (n=42); bedaquiline-linezolid 8-week regimen (n=189). All 4 regimens had lower efficacy than the standard 24-week treatment regimen. The two high-dose rifampicin regimens had similar overall rates of severe and serious adverse events to standard treatment, consistent with previous high-dose rifampicin, although any evidence of excess of severe hepatotoxicity compared tom other trials was found.
Meya et al. NEJM. **Trial of high-dose oral rifampin in adults with tuberculous meningitis.** *NEJM.* 2025. [52]	Double-blind, randomized, placebo-controlled international clinical trial involving adults with TB meningitis, consisting in a 9-12 months standard daily isoniazid, rifampin (at a dose of 10 mg/kg/day, ethambutol, and pyrazinamide plus either additional rifampin (for a cumulative dose of 35 mg per kilogram; high-dose group) or matched placebo (standard-dose group) for 8 weeks. The study enrolled 499 patients. No evidence of beneficial effect from high-dose rifampin was observed, and the potential for a harmful effect cannot be established.
Sester et al. **Diagnostic accuracy and predictive value of the QuantiFERON-TB gold plus assay for tuberculosis in immunocompromised individuals: a prospective TBnet study.** *Lancet Reg Health Eur*. 2025. [32]	Prospective multinational observational study, enrolling 2,663 immunocompromised adults including people living with HIV (PLHIV), chronic renal failure, rheumatoid arthritis, solid-organ transplantation or stem-cell transplantation, and immunocompetent adults with and without TB-disease. Individuals without TB-disease were followed up for the development of TB. Among patients with chronic renal failure, rheumatoid arthritis, solid-organ transplantation or stem-cell transplantation, no-one developed active TB. In contrast, among 642 PLHIV without TB preventive treatment (TPT), one with an indeterminate QuantiFERON+ and 3/30 individuals with a positive QuantiFERON+ developed active TB.
Ning et al. **Self-powered rapid antigen-specific T-cell response assay for Mycobacterium tuberculosis infections.** *Nat Biomed Eng*. 2025. [102]	Description of a microfluidic chip-based antigen-specific T-cell response assay (ASTRA) that automates the detection of MTB-specific T-cell activation responses to facilitate screening for latent MTB infection and TB. Compared with IGRA, ASTRA shows greater diagnostic sensitivity in individuals with HIV-1 co-infections (93.8% vs 67%), comparable diagnostic sensitivity in HIV-negative individuals (92.8%) and faster detection (4 hours vs 24-48 hours). ASTRA holds the potential to facilitate efforts to control the global TB epidemic and serve as a versatile platform for analyzing T-cell responses across various infectious diseases and immunotherapeutic interventions in low resource countries.
Chesov et al. **High rates of acquired resistance to fluoroquinolones, bedaquiline, and linezolid in patients failing treatment against drug-resistant tuberculosis in the Republic of Moldova.** *CMI.* 2025. [7]	Retrospective cohort study analyzing national TB surveillance data (2021-2022) on patients diagnosed with MDR/RR-TB with available baseline and follow-up drug susceptibility testing for WHO group A drugs in the Republic of Moldova. The study included 1,034 patients initiating MDR/RR-TB treatment; 67.1% were reported as successfully treated. Baseline resistance to WHO group A drugs was significantly higher in patients with treatment failure than in those with successful outcomes: fluoroquinolones (66.7% vs 18.3%), bedaquiline (12.5% vs 0.6%), and linezolid (25.0% vs 0.6%). Acquired resistance occurred in 39.6% of those failing treatment, but none with successful outcomes, particularly to bedaquiline (30.9%), linezolid (16.7%), and fluoroquinolones (25.0%). Baseline fluoroquinolone resistance and acquired resistance to any WHO group A drug were associated with treatment failure. High rates of baseline and acquired drug resistance to key second-line anti-TB drugs as a driver for treatment failure in MDR/RR-TB.
Kherabi et al. **Treatment outcomes of extensively drug-resistant tuberculosis in Europe: a retrospective cohort study.** *Lancet Reg Health Eur*. 2025. [56]	Observational, retrospective cohort study included patients diagnosed with extensively drug-resistant TB in the WHO European Region from 2017 to 2023. Among 11,003 patients with rifampicin-resistant TB, 1.7% from 16 countries had extensively drug-resistant TB. Of these, 48.4% harbored strains with resistance to bedaquiline, 34.0% to linezolid, and 17.6% to both. In patients with unsuccessful treatment outcomes, most experienced treatment failure. Compared with other levels of drug resistance, treatment outcomes were significantly worse for extensively drug-resistant TB.
Sinha et al. **A roadmap for integrating nutritional assessment, counselling, and support into the care of people with tuberculosis.** *Lancet Glob Health*. 2025. [13]	A proposition for a comprehensive roadmap for integrating nutritional assessment, counseling, and support into TB treatment. Authors recommend standard nutritional assessment with anthropometric measurements, in addition to macronutrient and micronutrient support alongside nutritional counseling, weight monitoring during treatment. At the end of treatment, a reassessment of anthropometric measures to assess nutritional recovery is recommended. People with TB who remain underweight should receive close follow-up to detect early relapse.

**Table 5:** Future Priorities

Topic	Proposed three main research priorities in the field
Epidemiology	More collaborative modeling platforms to understand the future effect of global change and potential intervention effect on tuberculosis endpoints Clearer understanding of the mortality and morbidity reduction after clinical and treated TB in population cohorts Better assessment of population attributable risks for comorbidities in TB and projection of their dynamic impact on a population scale
Comorbidities	Identify comorbidity-specific determinants of poor TB outcomes. Strengthen predictive models to guide targeted screening and resource allocation. Evaluate therapeutic strategies that improve outcomes in comorbid TB.
Pathogenesis and Prevention	Need for biomarkers with better predictive value for progression. Better identification of patients without risk for progression among immunocompromised patients. Need for better understanding of correlates of protection and progression to be implemented in vaccine design.
Diagnostic	Development and validation of biomarkers predictive of TB progression, to identify individuals at increased risk of progression from infection or asymptomatic TB to active disease, including in key risk groups. Focus research on possible diagnostic tools for the detection of asymptomatic TB, enabling earlier identification of disease along the TB spectrum. Alignment of microbiological diagnostics and drug susceptibility testing with new TB drugs and regimens, ensuring timely, actionable resistance detection that informs treatment selection and monitoring.
Treatment	Less intensive treatment regimens trialed for lymph-node TB and other paucibacillary disease (not necessarily shorter treatment). The experience on treatment of people affected by TB and treatment effect of novel regimens on health-related quality of life, including which tools are most appropriate to measure. Long-acting injectable agents in TB disease evaluated in pragmatic trials to investigate real-world effectiveness and risk of resistance developing.
Post-TB lung disease	Definition of post-TB outcomes and patient related outcomes and inclusion thereof in clinical trials of new TB drugs and host directed therapies in TB. In depth research in TB prevention and early diagnosis and its impact on the long-term trajectories of post TB outcomes. Development of strategies and implementation research on integration of post-TB care in programmatic management of TB and chronic lung health
Pediatrics	Improved immune-based tests for the detection of TB disease with robust performance in children. Research into host biomarkers of TB infection and disease that achieve high sensitivity and specificity in children and adolescents. Interventions to improve the outcome of TB meningitis in children & adolescents.
NTM	Elucidate immune mechanisms underlying susceptibility and protection of NTM lung disease. Identify biomarkers to predict who will benefit most from antimicrobial therapy/who is at highest risk of NTM lung disease progression. Evaluate novel antimicrobial *M. avium/M. abscessus* complex treatment regimens and non-pharmacological approaches in clinical trials.
